# Association of Anticholinergic Use with Incidence of Alzheimer’s Disease: Population-based Cohort Study

**DOI:** 10.1038/s41598-019-43066-0

**Published:** 2019-05-01

**Authors:** Kyung-in Joung, Sukil Kim, Yoon Hee Cho, Sung-il Cho

**Affiliations:** 1 Division of Epidemiology, Department of Public Health Science, Graduate School of Public Health, Seoul National University, 08826 Seoul, Republic of Korea; 2 Department of Preventive Medicine, College of Medicine, The Catholic University of Korea, 06591 Seoul, Republic of Korea; 3 Department of Biomedical & Pharmaceutical Sciences, The University of Montana, 59812 Missoula, USA; 4 Department of Public Health Science, Graduate School of Public Health and Institute of Health and Environment, Seoul National University, 08826 Seoul, Republic of Korea

**Keywords:** Risk factors, Alzheimer's disease, Epidemiology

## Abstract

Drugs with strong anticholinergic properties are used under a variety of conditions; however, they can cause various adverse effects including a negative impact on cognitive functions, with older adults being more susceptible to these effects. We explored whether the use of anticholinergic agents (ACs) affects the risk of Alzheimer’s disease (AD) in terms of incidence by using National Health Insurance Service elderly cohort database (2002–2013). As a result, AD risk was higher in subjects with an increased amount of prescriptions for strong ACs over a long period of time (9–12 years) than that in the least-exposed reference group (0–9 dose/year) [hazard ratio (HR) (95% confidence interval (95% CI)) 0.99 (0.95–1.03), 1.19 (1.12–1.26), 1.39 (1.30–1.50); in the 10–49 doses/year, 50–119 doses/year, and ≥120 doses/year groups]. Hazard ratios were particularly high in the young-old subgroup (60–64 years old in 2002) [HR (95% CI) 1.11 (1.04–1.22), 1.43 (1.25–1.65), 1.83 (1.56–2.14); in the 10–49 doses/year, 50–119 doses/year, and ≥120 doses/year groups]. Use of strong ACs dose-dependently increased the risk of AD in terms of incidence when exposure was followed up for 9 years or more, and the association was greater in the young-old subgroup.

## Introduction

Drugs with anticholinergic properties are used in a variety of conditions such as depression, sleep disorder, allergic conditions, and Parkinson’s disease. Despite their wide usage and efficacy, anticholinergic agents (ACs) can cause various adverse systemic effects, as muscarinic acetylcholine receptors (M1–M5) are distributed in diverse organs including the central nervous system^1^. Older adults are especially susceptible to the side effects of ACs, attributable to an overall decrease in organ function as well as lowered choline uptake in the brain^2^. Hence, many ACs are potentially inappropriate medications (PIMs) for older adults^3^. Numerous studies have demonstrated that strong ACs are negatively associated with cognitive functions in older people^4–11^, with few exceptions^12^. However, whether their use affects the risk of AD in terms of incidence is controversial, and studies are limited. Some cohort studies and two case-control studies of older adults reported from 1.1 to 3.4 fold increases in the incidence of dementia among ACs users differentiated by adopted scale, follow-up period, and level of exposure, etc.^13–18^; however, other studies did not show the relevance^9,10^.

Anticholinergic properties are generally quantified by the combination of a serum radioreceptor anticholinergic activity assay (SAA)^19^, *in vitro* measurement of affinity to muscarinic receptors^20^, or expert opinions^21^. Several investigators have published lists of ACs, and most of them graded the ACs into 2–4 levels according to their anticholinergic potency^21–25^. Although studies of anticholinergic medications usually select one of these validated anticholinergic rating scales, the methods to quantify their exposure are varied and only a few studies quantitatively measured their exposure up to the actual amount. Among the previous studies examining the association between anticholinergic use and risk of dementia, only two studies elaborated on the methods used to measure their use^13,17^; the others assessed them by their use at any time prior to the diagnosis of dementia^14^, medications during the preceding month^15^, or methods were not described in detail^10^. Furthermore, ACs with a level 1 anticholinergic rating (considered as a weak, possible or indefinite AC, such as ranitidine and atenolol) are generally quantified by merging with strong or definite ACs in many studies of health outcomes associated with anticholinergic use. However, these agents only show anticholinergic activity *in vitro*, which is not clinically meaningful^25^. Further, several studies reporting the negative impact of strong ACs on cognitive functions failed to find the relevance of those weak ACs with mild cognitive impairment (MCI)^12,26^. Therefore, it would be more appropriate to assess the effects of weak ACs separately from strong or definite ACs when examining the association between ACs and AD.

Age is the greatest non-modifiable risk factor of AD^27^. It is unlikely that elderly people of all ages will be affected on an equal level by protective measures or risk factors. From a preventive point of view, the young-old population with a longer life span, such as elderly people under 65, especially needs to be assessed for the impact of ACs on the risk of AD; however, none of the existing studies have focused on the young-old population.

The preclinical stage before onset of the clinical phenotype is long^27^. Complete AD may arise more than 15 years after the initial detection of a positive marker^28^. While ‘short-term use of ACs prior to the onset of disease’ could be an appropriate variable of interest in studies on incidence of acute disease impairments such as delirium^7,16,29^, it seems more reasonable to assume that the incidence of dementia is affected by long-term rather than short-term exposures. However, only two longitudinal studies have been evaluated for long-term exposure over 5 years, and they reported conflicting results^10,13^. In addition, one of the greatest challenges to investigating the relationship between anticholinergic exposure and risk of AD involves the exclusion of prodromal effects. In the prodromal state prior to the development of dementia, use of anticholinergic antidepressants or antipsychotics may increase because of prodromal symptoms—leading to protopathic bias^30^. In observational studies to elucidate the causal relationship between anticholinergic use and risk of AD, this protopathic bias should be excluded as much as possible; however, very few studies have done so^13,17^.

Therefore, we investigated whether AC use increases the risk of AD in terms of incidence in a large population representing a Korean older population. This study complemented some limitations of existing cohort studies; long-term anticholinergic exposure was completely quantified for individuals, and the effects of strong ACs and weak ACs were examined separately. We also looked closely at the relationship in the subgroup of relatively young-old people (60–64 years old in 2002). Varied analyses to exclude prodromal bias were also conducted.

## Methods

### Data source

We used the National Health Insurance Service (NHIS) elderly cohort, which is a population-based cohort established by the NHIS in South Korea. This cohort includes detailed information regarding medical utilization of about 550,000 elderly people aged 60–119 as of 2002 corresponding to about 10% of the total eligible Korean elderly population in 2002, who were followed for 11 years until 2013, unless participants’ eligibility was disqualified due to death or emigration^31,32^. The National Health Insurance (NHI) is a single-insurer system having complete universal healthcare coverage in Korea since 2000^31^. The medical-treatment DB includes details of electronic medical-treatment bills, diagnoses, prescriptions, etc. Details about the DB can be obtained from the NHI Sharing Service website (https://nhiss.nhis.or.kr/bd/ab/bdaba015lv.do). Information regarding all medicines licensed and distributed in Korea was obtained from the Health Insurance Review and Assessment Service (HIRA), a government-affiliated organization that reviews and assesses healthcare costs and healthcare service quality, as well as supporting the NHI policy in determining medical fee schedules and drug prices^33^.

### Study subjects and follow-up

**Figure 1:**
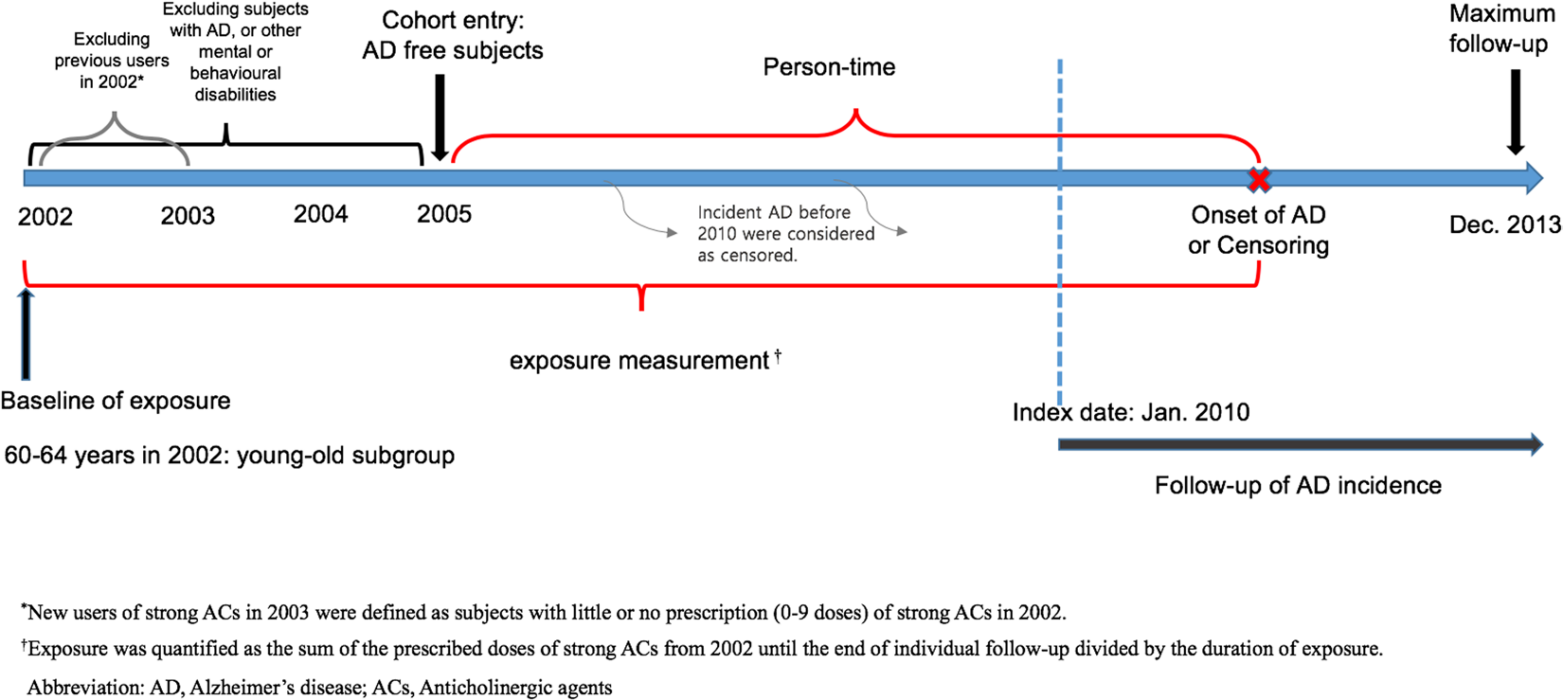
Schematic representation of participants selections and follow-up.

We recruited 367,871 people from the older population without diagnosis of a mental or behavioural disability (International Classification of Diseases-10 (ICD-10): F00-F99) or AD (ICD-10: G30) as a primary or secondary diagnosis for 3 years (2002–2004) for this study. Among them, 255,222 new users of strong ACs in 2003 were followed up for incident AD from 2005 to 2013. New users of strong ACs in 2003 were operationally defined as subjects with few or no prescriptions for strong ACs in 2002; the criteria for new users included those who did not use strong ACs at all in 2002, as well as those who were prescribed strong ACs but only filled a few prescriptions (1–9 doses/year), as some ACs such as chlorpheniramine are widely used in a variety of diseases, including common cold and allergies. Since we targeted long-term exposure and a proportional hazard was observed by the log-negative-log Kaplan–Meier plot after about 6 years of person-time starting from 2005, only 191,805 subjects whose follow-up end date was in 2010 or later were finally included, as illustrated in Fig. 1.

During the follow-up, people with a diagnosis of AD (ICD-10: F00 or G30) and a prescription for anti-AD agents (i.e., donepezil, rivastigmine, galantamine, or memantine) at the time of diagnosis were defined as AD patients, and the first day was defined as the incident date. In the censored cases, the follow-up period was defined as the period from 2005 to the date of death or the date of their last visit to a medical facility, whichever occurred first. Subjects 60–64 years old in 2002 were defined as the “young-old” subgroup, and major analyses were conducted for this subgroup along with all subjects. In this young-old subgroup, AD cases occurred at or less than 75 years old.

### Selection of anticholinergic agents and measurement of their exposure

Selection and classification of anticholinergic agents were based on the Beers Criteria^24^ and Anticholinergic Cognitive Burden (ACB) scale^25^. The AGS (American Geriatric Society) Beers criteria is a composite of several scales including the ACB scale. However, the ACB scale was combined with the Beers criteria in this study after reviewing other frequently referenced scales^10,12,21–23^; the Beers criteria only provides a list of strong ACs, and the ACB scale is thought to be the only scale to focus on reflecting cognitive burden among those scales. Drugs listed in the Beers Criteria as strong anticholinergic agents (strong ACs) or with an ACB score of 2 or 3 were classified as “strong ACs”, while drugs with an ACB score of 1 were defined as “weak ACs”. Supplementary Tables S1 and S2 provide the lists of them. Strong ACs have been classified as potentially inappropriate for the elderly due to the high risk of various adverse effects in this population^24^. Exposure to ACs was measured by calculating standardized prescribed doses. Generic name code, dosage unit, daily frequency of administration, and number of prescribed days in the NHIS data were used. The minimum daily dose for adults for each anticholinergic agent was set based on the Korean drug formulary. Supplementary Table S3 provides the dose. The standardized prescribed doses of ACs from 2002 until the end of individual follow-up were summed and divided by the period to calculate the prescribed amount of ACs. The formula for obtaining the average annual prescribed amount was as follows:

$$\begin{array}{rcl}\cdot \,{\rm{Standardized}}\,{\rm{prescribed}}\,{\rm{doses}}\,({\rm{doses}}) & = & {\rm{strength}}\,{\rm{in}}\,{\rm{prescribed}}\,{\rm{drug}}\\  &  & /\,{\rm{minimum}}\,{\rm{daily}}\,{\rm{dosage}}\,{\rm{for}}\,{\rm{adult}}\\  &  & \times \,{\rm{number}}\,{\rm{of}}\,{\rm{unit}}\,{\rm{of}}\,{\rm{administration}}\\  &  & \times \,{\rm{daily}}\,{\rm{frequency}}\,{\rm{of}}\,{\rm{administration}}\\  &  & \times \,{\rm{number}}\,{\rm{of}}\,{\rm{prescribed}}\,{\rm{days}}\end{array}$$

$$\begin{array}{rcl}\cdot \,{\rm{Prescribed}}\,{\rm{amount}}\,({\rm{doses}}/{\rm{year}}) & = & \sum \,{\rm{standardised}}\,{\rm{prescribed}}\,{\rm{doses}}\\  &  & /{\rm{observed}}\,{\rm{period}}\,{\rm{from}}\,{\rm{2002}}\end{array}$$

Subjects were divided into four classes according to the prescribed amount of strong ACs (0–9 doses/year, 10–49 doses/year, 50–119 doses/year, ≥120 doses/year). For weak ACs, the subjects were grouped into four classes by their prescribed amount (0–29 doses/year, 30–119 doses/year, 120–299 doses/year, ≥300 doses/year) and the same analyses were performed.

### Statistical analyses

To identify and control for potential confounding variables, basic demographic variables such as age, sex, income level; Charlson comorbidity index (CCI); medical histories such as hypertension (HTN), diabetes mellitus (DM), myocardial infarction (MI), cardiovascular disease (CVD), Parkinson’s disease (PD), epilepsy, neuralgia, dizziness including that due to vestibular abnormalities, sleep disorders, genitourinary diseases, and skin diseases; and use of weak ACs and of other non-ACs that can impair cognitive function were included as covariates. Records of ICD-10 diagnostic codes in 2008 were used as surrogates for information on medical histories. In detail, subjects diagnosed with HTN, DM, MI, or CVD, which are the main diseases in the elderly, as well as strong AC-indicated diseases such as PD, epilepsy, neuralgia, dizziness, respiratory diseases, sleep disorders, genitourinary diseases, and skin diseases as primary or secondary diagnoses at least three times in 2004 were considered as having the corresponding medical histories. The ICD-10 codes used to define each disease (group) are presented in Supplementary Table S4. The use of “other non-ACs that can impair cognitive function” was determined using AGS Beers Criteria and the list are presented in Supplementary Table S5.

A proportional hazard regression model was used to assess the association between anticholinergic use and incidence of AD. To verify proportional hazard assumptions, the log-negative-log of the Kaplan–Meier estimator of the survival function was applied.

### Supplementary analyses

Use of strong ACs may be attributable to prodromal symptoms of AD before the first diagnosis, as prescribed amount of strong ACs was calculated after excluding those during the 1 and 2 years prior to the end of follow-up. As depression is highly prevalent in the prodromal stage of AD, analysis was carried out after excluding “anticholinergic antidepressants”. For drug subclasses with high prescribed amount among all strong ACs, including antihistamines, antidepressants, and antimuscarinics, the prescribed amounts were also calculated separately, and the association of exposure to each drug subclass with risk of AD was examined. Antipsychotic drugs could be used in the prodromal stage but were excluded from the analysis because of their low frequency of use.

### Sensitivity analysis

We also compared the results of continuous users and intermittent users. Continuous users included those who were not exposed to the lowest level (0–9 doses) during any one year of the follow-up period; the others were defined as intermittent users. Results for continuous users were assumed to be attributable to exclusion of those who used large amounts of strong ACs just before the onset of AD, resulting in a further exclusion of the protopathic bias. Thus, if the results in the continuous users are similar to those in all subjects, or if the effect size is larger, this would support the validity of this study.

All data were processed and analysed using SAS 9.4 (SAS Institute, Inc., Cary, NC).

### Ethics approval and consent to participate

The study has been approved by the Bioethics Committee of Seoul National University Institutional Review Board (IRB) and the approval number is E1705 / 001-003. IRB deemed this study exempt from approval and waived the need for informed consent.

## Results

### Descriptive data

Among 191,805 subjects, 96,008 (50.1%) rarely used strong ACs (0–9 doses/year), while 24,542 (12.8%) were exposed to large amounts of ACs with more than 50 doses per year. Very large amounts of strong ACs (≥120 doses/year) were prescribed in 8,686 (4.5%) subjects. Antihistamines, antidepressants, and urinary antimuscarinics were the most prescribed among strong anticholinergic drug classes (Table 1).

**Table 1:** General characteristics of study subjects by prescribed amount of strong anticholinergic agents.

	Prescribed amount of strong anticholinergic agents (doses/year) (n = 191,805)			
	0–9	10–49	50–119	≥120
No. of subjects (%)	96,008 (50.1)	71,255 (37.2)	15,856 (8.3)	8,686 (4.5)
Sex, male	45,933 (47.8)	31,019 (43.5)	6,960 (43.9)	3,855 (44.4)
Age (years)	66.8 ± 6.2	66.6 ± 5.7	67.5 ± 5.8	67.6 ± 5.7
Person-year	3139.8 ± 257.6	3179.2 ± 234.5	3148.8 ± 272.1	3135.5 ± 280.8
**Level of income**				
Medical aid	7,416 (7.7)	9,760 (13.7)	4,247 (26.8)	3,053 (35.2)
4/10 percentile	27,088 (28.2)	19,114 (26.8)	3,908 (24.7)	1,859 (21.4)
8/10 percentile	34,298 (35.7)	24,211 (34.0)	4,439 (28.0)	2,081 (24.0)
10/10 percentile	27,206 (28.3)	18,170 (25.5)	3,262 (20.6)	1,693 (19.5)
**Strong ACs** ^*^				
Mean ± SD	4.0 ± 2.9	22.3 ± 10.4	75.8 ± 19.4	241.8 ± 156.2
Median [IQR]	3.6 [1.4–6.3]	19.3 [13.8–28.8]	71.7 [58.8–90.3]	189.8 [146.2–277.8]
**Weak ACs** ^*^				
Mean ± SD	50.5 ± 124.0	74.8 ± 135.4	109.4 ± 158.5	144.4 ± 189.9
Median [IQR]	7.2 [2.0–34.4]	25.3 [9.4–78.3]	56.8 [21.9–133.3]	81.4 [28.1–187.9]
Antihistamines^*^	3.3 ± 2.7	16.9 ± 10.1	46.7 ± 30.6	83.7 ± 104.2
Antidepressants^*^	0.2 ± 0.7	2.0 ± 5.4	13.0 ± 23.7	91.0 ± 150.9
Urinary antimuscarinics^*^	0.1 ± 0.7	1.5 ± 4.9	9.2 ± 21.2	31.8 ± 73.0
Other non-anticholinergic agents that can impair cognitive function^†^	6.6 ± 23.4	14.3 ± 33.8	28.9 ± 52.3	46.2 ± 75.8
**Charlson Comorbidity Index**				
0–1	47,749 (50.0)	20,295 (28.5)	20,295 (16.7)	1,138 (13.1)
2–3	37,509 (39.1)	39,890 (56.0)	9,788 (61.7)	5,109 (58.8)
≥4	10,750 (11.2)	11,070 (15.5)	3,421 (21.6)	2,439 (28.1)
**Medical histories (%)**				
Hypertension	19,045 (19.8)	16,624 (23.3)	3,466 (21.9)	1,812 (20.9)
Cardiovascular disease	1,973 (2.1)	1,533 (2.2)	518 (3.3)	355 (4.3)
Myocardial infarction	858 (0.9)	722 (1.0)	185 (1.2)	95 (1.1)
Diabetes mellitus	7,291 (7.6)	6,284 (8.8)	1,495 (9.4)	902 (10.4)
Parkinson’s disease	41 (0.0)	32 (0.0)	19 (0.1)	65 (0.8)
Epilepsy	79 (0.1)	73 (0.1)	26 (0.2)	46 (0.5)
Neuralgia	1,486 (1.6)	2,351 (3.3)	629 (4.0)	332 (3.8)
Dizziness	897 (0.9)	1,216 (1.7)	409 (2.6)	214 (2.5)
Sleep disorder	123 (0.1)	176 (0.3)	63 (0.4)	43 (0.5)
Genitourinary disease	1,463 (1.5)	2,004 (2.8)	663 (4.2)	485 (5.6)
Skin disease	1,386 (1.4)	2,566 (3.6)	742 (8.3)	433 (4.5)

All values were presented with mean ± SD, median [IQR] or frequency (%).

^*^Average annual prescription amount from 2002 to end of the follow-up (unit. Doses/year).

^†^Calculated as average annual cumulative prescribed days from 2002 to end of the follow-up.

Abbreviation: ACs, anticholinergic agents; IQR, interquartile range.

AD occurred in 13,133 (6.9%) cases. Compared to censored subjects, more AD incident subjects received ≥50 doses/year of strong ACs (12.3% vs. 19.4%). Antihistamines, antidepressants, and antimuscarinics (in that order) were the most prescribed drug classes in both groups, and incident AD subjects were prescribed more prescriptions for all these drug subclasses. The proportion of subjects receiving ≥120 doses/year of weak ACs was also higher in the AD incident subjects, but not as significantly different as in the strong ACs (16.3% vs. 18.9%) (Table 2).

**Table 2:** Characteristics of study subjects in Non-incident Alzheimer’s disease group and incident Alzheimer’s disease group.

	Non-incident Alzheimer’s disease	Incident Alzheimer’s disease
No. of subjects (%)	178,672 (93.1)	13,133 (6.9)
Sex, male	83,404 (46.7)	4,363 (33.2)
Age (years)	66.5 ± 5.8	70.6 ± 6.5
**Level of income**		
Medical aid	21,163 (11.8)	3,313 (25.2)
4/10 percentile	48,822 (27.3)	3,147 (24.0)
8/10 percentile	61,420 (34.4)	3,609 (27.5)
10/10 percentile	47,267 (26.5)	3,064 (23.3)
Person-year (day)	3184.4 ± 220.4	2755.3 ± 312.9
**Strong ACs** ^*^	26.7 ± 59.4	38.6 ± 80.3
0–9	90,145 (50.5)	5,863 (44.6)
10–49	66,541 (37.2)	4,714 (35.9)
50–119	14,353 (8.0)	1,503 (11.4)
≥120	7,633 (4.3)	1,053 (8.0)
**Weak ACs** ^*^	68.1 ± 136.9	76.2 ± 140.1
0–29	109,205 (61.1)	7,217 (55.0)
30–119	40,299 (22.6)	3,451 (26.3)
120–299	19,634 (11.0)	1,693 (13.0)
≥300	9,534 (5.3)	772 (5.9)
Antihistamines^*^	15.3 ± 30.6	19.2 ± 38.6
Antidepressants^*^	5.7 ± 36.6	10.2 ± 53.4
Urinary antimuscarinics^*^	2.7 ± 17.9	4.3 ± 22.5
Other non-anticholinergic agents that can impair cognitive function^†^	12.6 ± 34.9	20.0 ± 45.5
**Charlson Comorbidity Index**		
0–1	67,710 (37.9)	4,119 (31.4)
2–3	85,540 (47.9)	6,756 (51.4)
≥4	25,422 (14.2)	2,258 (17.2)
**Medical histories**		
Hypertension	38,381 (21.5)	2,566 (19.5)
Cardiovascular disease	4,035 (2.3)	344 (2.6)
Myocardial infraction	1,720 (1.0)	1,152 (1.1)
Diabetes mellitus	14,820 (8.3)	1,152 (8.8)
Parkinson’s disease	131 (0.1)	26 (0.2)
Sleep disorder	364 (0.2)	41 (0.3)
Dizziness	2,490 (1.4)	246 (1.9)
Genitourinary disease	4,326 (2.4)	289 (2.2)
Epilepsy	200 (0.1)	24 (0.2)
Neuralgia	4,424 (2.5)	374 (2.9)
Skin disease	4,790 (2.7)	337 (2.6)

All values were presented with mean ± SD, median [IQR] or frequency (%)

^*^Average annual prescription amount from 2002 to end of the follow-up (unit. Doses/year).

^†^Calculated as average annual cumulative prescribed days from 2002 to end of the follow-up.

Abbreviations: ACs, anticholinergic agents; IQR, interquartile range.

### Outcome, main results

A crude model and two covariate models were set up. In Model I, age, sex, and income level were included for adjustment, while in Model II, we adjusted for all 16 covariates outlined in the Methods section (Table 3).

**Table 3:** Risk of Alzheimer’s disease by prescribed amount of strong anticholinergic agents.

Subjects	Prescribed amount (doses/year)	No. of AD events (%)	Incidence rate per 100,000 person-years	Crude model	Covariates model	
					Model I	Model II
				Hazard ratio (95% Confidence interval)		
All subjects (n = 191,805)	overall	13,133 (6.9)	2.17 (2.13–2.21)			
	0–9	5,863 (6.1)	1.94 (1.90–1.99)	Reference	Reference	Reference
	10–49	4,714 (6.6)	2.08 (2.02–2.14)	1.03 (0.99–1.07)	1.02 (0.98–1.06)	0.99 (0.95–1.03)
	50–119	1,503 (9.5)	3.01 (2.86–3.16)	1.53 (1.44–1.61)	1.28 (1.21–1.36)	1.19 (1.12–1.26)
	≥120	1,053 (12.1)	3.87 (3.63–4.10)	1.98 (1.86–2.12)	1.57 (1.47–1.68)	1.39 (1.30–1.50)
Young-old subgroup (n = 84,566)	overall	2,542 (3.0)	0.94 (0.90–0.98)			
	0–9	1,032 (1.2)	0.73 (0.69–0.78)	Reference	Reference	Reference
	10–49	981 (3.1)	0.97 (0.90–1.03)	1.27 (1.17–1.39)	1.18 (1.08–1.29)	1.11 (1.04–1.22)
	50–119	295 (5.0)	1.56 (1.39–1.74)	2.09 (1.83–2.37)	1.70 (1.49–1.94)	1.43 (1.25–1.65)
	≥120	234 (7.6)	2.39 (2.09–2.70)	3.23 (2.81–3.73)	2.47 (2.14–2.87)	1.83 (1.56–2.14)

Model I: Adjusted for age, sex and level of income.

Model II: Adjusted for Model I + diabetes mellitus, hypertension, myocardial infarction, cardiovascular diseases, dizziness, genitourinary diseases, epilepsy, Parkinson’s disease, neuralgia, skin disease, sleep disorder, prescribed doses of weak anticholinergics, and cumulative prescribed days of other non-anticholinergic agents that can impair cognitive function (all psychotic diseases including depression, psychosis, anxiety were excluded in the step of participant selection in advance).

The hazard ratios (HRs) were significantly higher in subjects exposed to ≥50 doses/year of strong ACs than in the reference group in both the crude and two-covariate models [0.99 (0.95–1.03), 1.19 (1.12–1.26), 1.39 (1.30–1.50); in the 10–49 doses/year, 50–119 doses/year, and ≥120 doses/year groups, respectively; Model II]. In particular, the HRs were highest, and a clear dose response was observed in the young-old subgroup [1.11 (1.04–1.22), 1.43 (1.25–1.65), 1.83 (1.56–2.14); in the 10–49 doses/year, 50–119 doses/year, and ≥120 doses/year groups, respectively; Model II] (Table 3).

Results regarding whether exposure to weak anticholinergic drugs increased the risk of AD are presented in Table 4. HRs were higher in the groups with higher exposure in the crude model and in Model I. However, no associations were found in the final Model (Table 4).

**Table 4:** Risk of Alzheimer’s disease by prescribed amount of weak anticholinergic agents.

Subjects	Prescribed amount (doses/year)	No. of AD events (%)	Incidence rate per 100,000 person-years	Crude Model	Covariates Model	
					Model I	Model II
				Hazard ratio (95% Confidence interval)		
All subjects (n = 191,805)	overall	13,133 (6.9)	2.17 (2.13–2.21)			
	0–29	7,217 (6.2)	1.97 (1.92–2.01)	Reference	Reference	Reference
	30–119	3,451 (7.9)	2.49 (2.41–2.57)	1.24 (1.19–1.29)	1.09 (1.05–1.14)	1.01 (0.97–1.06)
	120–299	1,693 (7.9)	2.51 (2.39–2.63)	1.25 (1.19–1.32)	1.08 (1.03–1.14)	0.97 (0.92–1.03)
	≥300	772 (7.5)	2.38 (2.13–2.21)	1.21 (1.12–1.30)	1.09 (1.01–1.17)	0.95 (0.88–1.02)
Young-old subgroup (n = 84,566)	overall	2,542 (3.0)	0.94 (0.90–0.98)			
	0–29	1,358 (2.5)	0.78 (0.74–0.82)	Reference	Reference	Reference
	30–119	699 (3.9)	1.22 (1.13–1.31)	1.53 (1.39–1.67)	1.36 (1.24–1.49)	1.17 (1.06–1.29)
	120–299	326 (4.0)	1.24 (1.10–1.37)	1.56 (1.38–1.76)	1.37 (1.21–1.54)	1.09 (0.97–1.22)
	≥300	159 (4.1)	1.27 (1.07–1.47)	1.61 (1.37–1.90)	1.44 (1.22–1.69)	1.10 (0.93–1.31)

Model I: Adjusted for age, sex and level of income.

Model II: Adjusted for Model I + diabetes mellitus, hypertension, myocardial infarction, cardiovascular diseases, dizziness, genitourinary diseases, epilepsy, Parkinson’s disease, neuralgia, skin disease, sleep disorder, prescription amount of strong anticholinergics, and cumulative prescribed days of other non-anticholinergic agents that can impair cognitive function (all psychotic diseases including depression, psychosis, anxiety were excluded in the step of participant selection in advance).

### Supplementary analyses

Significant differences were maintained when the prescribed amount of strong ACs was calculated after excluding those prescribed during the 1 and 2 years prior to the end of follow-up. After excluding exposure to anticholinergic antidepressants, which are thought to have been used frequently for prodromal symptoms of AD, the risk of AD was still higher in the more-exposed group. When exposure was limited to drug subclass of antidepressants or antimuscarinics, which were more commonly used in AD patients, the estimation was similar to the main outcomes. When the exposure parameters were limited to antihistamines alone, which appears to be the subclass least related to the treatment of prodromal AD symptoms, the risk of AD was still significantly higher in the more-exposed group, even though the effect size was smaller than that of the antidepressants and urinary antimuscarinics [1.12 (1.05–1.19), 1.36 (1.29–1.44), 1.31 (1.24–1.38); in the exposed group to antihistamines, antidepressants, and urinary antimuscarinics, respectively; for all subjects, Model II]. Antipsychotic drugs also have a potential for protopathic bias in AD; however, these drugs were not analysed in this study because of low usage (Table 5).

### Sensitivity analyses

Continuous users had a higher risk of AD than intermittent users, supporting the robustness of this study as determined using the covariates Model II. As with main outcomes for all subjects, there was an increased risk of AD in the groups receiving 50–119 doses/year and the group receiving ≥120 doses/year than in the reference group. A dose response also appeared between these two exposure groups (Table 6).

**Table 5:** Risk of Alzheimer’s disease by various exposure criteria of strong anticholinergic agents in the all subjects and young-old subgroup.

Prescribed amount* (doses/year)		All subjects (n = 191,805)		Young-old subgroup (n = 84,566)	
		No. of AD events (%)	HR (95% CI)	No. of AD events (%)	HR (95% CI)
Total strong anticholinergic agents- recent 1 year^†^	0–9	6,327 (6.3)	Reference	1,137 (2.5)	Reference
	10–49	4,539 (6.6)	0.95 (0.92–0.98)	941 (3.1)	1.05 (0.96–1.15)
	50–119	1,351 (9.4)	1.13 (1.06–1.20)	259 (4.9)	1.31 (1.13–1.51)
	≥120	916 (11.9)	1.32 (1.22–1.42)	208 (7.7)	1.74 (1.47–2.05)
Total strong anticholinergic agents- recent 2 year^‡^	0–9	8,019 (6.3)	Reference	1,508 (2.6)	Reference
	10–49	3,448 (7.2)	0.99 (0.95–1.03)	689 (3.4)	1.04 (0.95–1.14)
	50–119	881 (9.6)	1.03 (1.10–1.19)	185 (5.7)	1.35 (1.15–1.58)
	≥120	785 (11.5)	1.23 (1.14–1.33)	160 (6.6)	1.37 (1.15–1.63)
Total strong anticholinergic agents- antidepressants	0–24	9,449 (6.3)	Reference	1,808 (2.6)	Reference
	25–59	2,104 (8.1)	1.10 (1.05–1.15)	430 (4.0)	1.20 (1.07–1.33)
	≥60	1,580 (10.5)	1.24 (1.18–1.32)	304 (5.9)	1.41 (1.23–1.61)
Antihistamines	0–49	11,898 (6.6)	Reference	2,339 (2.9)	Reference
	≥50	1,235 (10.1)	1.12 (1.05–1.19)	203 (5.0)	1.09 (0.94–1.27)
Antidepressants	0–9	11,668 (6.6)	Reference	2,165 (2.7)	Reference
	≥10	1,465 (10.8)	1.36 (1.29–1.44)	377 (6.9)	1.74 (1.55–1.96)
Urinary antimuscarinics	0–2	11,565 (6.7)	Reference	2,222 (2.8)	Reference
	≥3	1,568 (9.6)	1.31 (1.24–1.38)	320 (5.0)	1.42 (1.26–1.61)

^*^Average annual prescribed amount from 2002 to the end of follow-up period.

^†^Recent 1 year: prescribed amount during 1 year prior to the end of the follow-up.

^‡^Recent 2 years: prescribed amount during 2 years prior to the end of the follow-up.

Abbreviation: AD, Alzheimer’s disease.

**Table 6:** Risk of Alzheimer’s disease by prescribed amount of strong anticholinergic agents among continuous users and intermittent users.

Prescribed amount (doses/year)	Continuous users			Intermittent users		
	No. of subjects	No. of AD events (%)	Hazard ratio (95% CI)	No. of subjects	No. of AD events (%)	Hazard ratio (95% CI)
0–9^*^	96,008	5,863 (6.1)	Reference	96,008	5,863 (6.1)	Reference
10–49	1,499	73 (4.9)	0.84 (0.67–1.06)	69,756	4,641 (6.7)	0.99 (0.95–1.03)
50–119	1,404	109 (7.8)	1.20 (0.99–1.45)	14,452	1,394 (9.7)	1.19 (1.17–1.27)
≥120	902	90 (10.0)	1.50 (1.21–1.85)	7,784	963 (12.4)	1.38 (1.29–1.59)

Adjusted for sex, age, level of income, diabetes mellitus, hypertension, myocardial infarction, cardiovascular diseases, dizziness, genitourinary diseases, epilepsy, Parkinson’s disease, neuralgia, skin disease, sleep disorder, prescription amount of strong anticholinergics, and cumulative prescribed days of other non-anticholinergic agents that can impair cognitive function (all psychotic diseases including depression, psychosis, anxiety were excluded in the step of participant selection in advance).

^*^Subjects belonging to the 0–9 doses/year were used as references in both the continuous and intermittent user groups.

## Discussion

Using the NHIS elderly cohort DB for 2002–2013, we found that greater use of strong ACs for long periods (9 years or more) increased the risk of AD in terms of incidence, and the association was greater in young-old adults. These findings support previous studies indicating that long-term use of potent ACs is associated with incident dementia^13,17^. The extent of risk for AD in this study was comparable to that reported by Gray *et al*.^13^ in which the group most exposed to strong ACs for 10 years had a 63% higher risk of AD than the lowest-exposed group^13^. The results of various supplementary analyses, such as excluding recent exposures and analysis by drug subclass, are in accordance with those from the most recent case-control study in which exposures in the four years before the index date were excluded to avoid protopathic bias. In that study, the adjusted odds ratio (95% CI) for average ACB scores > 5.0 was 1.11 (1.08–1.14), and both antidepressant and urological subcategories of strong ACs were associated with risk of incident dementia^17^.

Overall, there was little difference in the risk of AD between the lowest exposure control group (0–9 doses/year) and the next lowest exposure group (10–49 doses/year); this is in accordance with the results from Gray *et al*.^13^ in which an increased risk of incident dementia was not found in the next lowest exposure group. However, the risk of AD increased in groups exposed to ≥50 doses/year, and the hazard was particularly high in elderly patients prescribed ≥120 doses/year, showing a dose response in these two exposure groups. Approximately 13% of the elderly were exposed to ≥50 doses/year of strong ACs, implying that many older people are at risk of developing AD following an increased use of strong ACs.

The period of anticholinergic exposure associated with development of AD is not yet clear. Studies in which anticholinergic use was measured for 6 years did not show an association with the incidence of dementia^9^, while a 10-year long-term exposure study reported an increased risk of dementia^13^. This study, which determined relevance when long-term exposure was measured for more than 8 years, supports these previous studies; we tested the link between exposure to ACs ≤ 8 years and the risk of AD in this study; however, no relationship was found.

When the same analysis was conducted for the young-old subgroup only, the risk of AD from use of strong ACs was consistently greater than that for all subjects. Reasons for the greater relevance in the young-old are unknown. Medications with adverse effects on cognitive functions may have a greater impact on the expression of AD phenotypes in young-old subjects, as they may be less affected than old-old subjects by age itself and underlying diseases. In a study of longitudinal rates of change as a function of baseline age for AD individuals, the rate of decline in AD decreased with age^34^. Since the younger elderly may have had a shorter prodromal period and fewer potential comorbidities and medications, the more solid result in this subgroup may further support the causal relationship. As the young-old people are more likely to be in the early stage of AD and to have a longer life span, they could benefit more from preventive intervention.

In this study, no association was found between weak ACs and the risk of AD, suggesting that they do not have a clinically negative effect on cognitive function to the extent of increasing the risk of AD in terms of incidence. These results are likely reliable, since they support results from studies exploring the effects of exposure to possible ACs on cognitive impairment in which a relationship was not found^9,26^. In addition, in the recent case-control study to examine the risk of incident dementia, the effect size was very small [OR(95% CI) = 1.05(1.01–1.10) and a dose response was not found in subjects who were exposed to the greatest amount of drugs with an ACB score of 1^17^.

This study had several advantages compared to previous studies. First, since it included a large population representing the entire older Korean population, the results are generalisable. Second, measurement of exposure was highly precise by calculating the standardized prescribed doses. Third, the study was the first cohort study to demonstrate that strong ACs, but not weak ACs, are associated with risk of AD using a separate analysis. Fourth, this was the first study to show that young-old people have a greater risk of AD, which is associated with increased use of strong ACs. Fifth, by excluding all elderly people with a diagnosis of mental or behavioural disorders as well as those with any type of dementia or cognitive-impairment disorder from the study, we determined that increased use of ACs intensifies the absolute risk of AD in terms of incidence. Finally, consistent significance in additional analyses performed to exclude the possibility of reverse associations with prodromal symptoms and in more robust results in the continuous subgroup further demonstrated the causal link between exposure and risk of AD. Although not shown in the results, the risk of AD in subgroups without PD, epilepsy, or sleep disorder, which would have any underlying link to the development of AD, was almost the same for all subjects—supporting the association of strong ACs and risk of AD.

This study had several limitations. First, as with other studies using health-insurance claims data, inaccuracy due to modification of diagnostic codes or misdiagnosis could not be excluded, even if such cases were rare. However, the accuracy of the data has been reported to be more than 90%, and many studies have reported the sensitivity around 70%^35,36^. Only about 9% of people with dementia-like symptoms are misdiagnosed as having dementia^37^. Second, this study did not include use of non-prescription drugs, and underestimation of anticholinergic exposure is possible. Third, although care was taken to control for other factors related to risk of AD, the likelihood of residual confoundings cannot be ruled out, since some variables known as risk factors of AD (such as family history and educational level) were not available in the source dataset and could not be controlled. We did not apply time varying covariates, which is also a limitation. However, the results from the analysis in which covariates at the start of the exposure (not shown here) in 2002 were very similar to these 2008-year covariates-adjusted results, and a previous study also stated that the effect of time varying covariates did not considerably affect their findings^17^. Fourth, we attempted to exclude inverse correlations by excluding people with mental or behavioural disorders, including MCI, from the cohort entry and varying the exposure criteria; however, the possibility may remain, as the preclinical and prodromal stages in AD are very long^38,39^. In this respect, it may be more appropriate to interpret the results of this study as “the use of strong ACs can increase the risk of AD in terms of incidence”. Increased use of ACs may also escalate the speed from preclinical or mild cognitive impairment to AD or its diagnosis. Reducing overuse of strong ACs in older adults could slow down or postpone the progression to clinical AD. Finally, we could not consider the interactions between the anticholinergic exposure and underlying diseases, which may be another research topic in the future.

This study showed that the impact of ACs on the aging brain may be more severe than previously thought by demonstrating that increased use of strong ACs intensifies the risk of AD in terms of incidence.

High use of strong ACs is prevalent in the Korean elderly population, and efforts should be made to reduce it. The robust association between strong ACs and risk of AD in the young-old subgroup indicates an even greater need to reduce anticholinergic use in these subjects. From the viewpoint of prevention, prescriptions and use of strong ACs need to be more strictly controlled in this population.

This study provides specific guidance for reducing inappropriate use of ACs. The prescribed amount of weak ACs was nearly double that of strong ACs, and yet these agents were not related to the risk of AD. Efforts to reduce use of strong ACs should be prioritized. Drug subclasses such as antihistamines, antidepressants, and antimuscarinics for urinary diseases accounted for a large proportion of the anticholinergic burden. If strategies can be developed to focus on the use of these agents, the anticholinergic burden could be effectively reduced. Exposure to strong ACs has been shown to be related to the incidence of AD only with long-term follow-up of 8 years or more, and the strength of the association was greater in continuous users. Regarding the risk of AD, special attention should be given to minimizing prolonged and continuous use of strong ACs.

Further studies regarding the effects of anticholinergic medications on the course of dementia are needed. It will be necessary to examine whether a diagnosis of dementia reduces inappropriate anticholinergic use. The reversibility of the risk of AD due to anticholinergic use should also be investigated by examining the effects of discontinuing ACs.

## Conclusion

An increased use of strong ACs for long periods was associated with a higher risk of AD. The risk was more significant in the young-old group. Strong ACs not only have a negative impact on cognitive function but can also affect the risk of AD in terms of incidence.

## Supplementary information

Supplementary Tables

## Data Availability

The datasets used and/or analysed during the current study are available from the corresponding author on reasonable request. Raw data can be obtained at National Health Insurance Data Sharing Service homepage (https://nhiss.nhis.or.kr/bd/ay/bdaya001iv.do) via on-line data request with a fee. Drug database can be obtained from HIRA website (https://www.hira.or.kr/dummy.do?pgmid=HIRAJ010000005001).
