# Supplementary material for: Association of Anticholinergic Use with Incidence of Alzheimer’s Disease: Population-based Cohort Study
Source: Sci Rep. 2019 May 1;9:6802. doi: 10.1038/s41598-019-43066-0 (PMC6494800; doi:10.1038/s41598-019-43066-0)
Supplement: Supplementary file 1 — Supplementary Tables [file 41598_2019_43066_MOESM1_ESM.docx]

**Association of Anticholinergic Use with Incidence of Alzheimer’s disease: Population-based Cohort Study**

Kyung-in Joung, Sukil Kim, Yoon Hee Cho, Sung-il Cho

Supplementary Table S1. List of strong anticholinergic agents.

| Antihistamines | Antipsychotics |
| --- | --- |
| Brompheniramine | Chlorpromazine |
| Carbinoxamine | Clozapine |
| Chlorpheniramine | Loxapine |
| Clemastine | Molindone |
| Cyproheptadine | Olanzapine |
| Dexbrompheniramine | Perphenazine |
| Dexchlorpheniramine | Pimozide |
| Dimenhydrinate | Quetiapine |
| Diphenhydramine | Thioridazine |
| Doxylamine | Trifluoperazine |
| Hydroxyzine | **Antimuscarinics** |
| Meclizine | Darifenacin |
| Triprolidine | Fesoterodine |
| **Antiparkinsonian agents** | Flavoxate |
| Benztropine | Oxybutynin |
| Trihexyphenidyl | Solifenacin |
| Amantadine | Tolterodine |
| **Skeletal muscle relaxants** | Trospium |
| Cyclobenzaprine | **Antispasmodics** |
| Orphenadrine | Atropine |
| **Antidepressants** | Belladonna alkaloids |
| Amitriptyline | Clidinium chlordiazepoxide |
| Amoxapine | Dicyclomine |
| Clomipramine | Homatropine |
| Desipramine | Hyoscyamine |
| Doxepin | Propantheline |
| Imipramine | Scopolamine |
| Nortriptyline | **Antiemetics** |
| Paroxetine | Prochlorperazine |
| Protriptyline | Promethazine |
| Trimipramine | **Anticonvulsants** |
| **Antiarrhythmic** | Carbamazepine |
| Disopyramide | Oxcarbazepine |

Supplementary Table S2. List of weak anticholinergic agents.

| Alimemazine | Fentanyl |
| --- | --- |
| Alverine | Furosemide |
| Alprazolam | Fluvoxamine |
| Atenolol | Haloperidol |
| Bupropion hydrochloride | Hydralazine |
| Captopril | Isosorbide |
| Chlorthalidone | Loperamide |
| Cimetidine hydrochloride | Metoprolol |
| Clorazepate | Morphine |
| Codeine | Nifedipine |
| Colchicine | Prednisone |
| Coumadin | Quinidine |
| Diazepam | Ranitidine |
| Digoxin | Risperidone |
| Dipyridamole | Theophylline |
| Disopyramide phosphate | Trazodone |
| Hydrocortisone | Triamterene |

Supplementary Table S3. Minimum daily doses for adult for anticholinergic agents licensed in Korea

| Generic name | Dose (mg) | Generic name | Dose (mg) | Generic name | Dose (mg) |
| --- | --- | --- | --- | --- | --- |
| Alprazolam | 2 | Cyproheptadine Orotate | 4 | Metoprolol Succinate | 48 |
| Alverine Citrate | 120 | Dexbrompheniramine Maleate | 6 | Molindone HCl | 15 |
| Amantadine HCl | 100 | Dexchlorpheniramine Maleate | 6 | Morphine Sulfate Hydrate | 20 |
| Amantadine Sulfate | 100 | Diazepam | 4 | Nifedipine | 30 |
| Amitriptyline HCl | 10 | Dicyclomine HCl | 30 | Nortriptyline HCl | 30 |
| Amitriptyline HCl S.R. Gr. | 10 | Dimenhydrinate | 150 | Olanzapine | 5 |
| Amoxapine | 25 | Diphenhydramine HCl | 75 | Oxcarbazepine | 600 |
| Atenolol | 50 | Diphenhydramine Citrate | 38 | Oxybutynin HCl | 5 |
| Belladonna Total Alkaloid | 200 | Dipyridamole | 75 | Paroxetine Hydrochloride Hydrate | 10 |
| Belladonna Ext. | 32 | Disopyramide Phosphate | 75 | Perphenazine | 12 |
| Belladonna Leaf Ext. | 1 | Doxepin HCl | 25 | Pethidine HCl | 150 |
| Belladonna Tinc. D4 | 225 | Doxylamine Succinate | 25 | Pimozide | 2 |
| Belladonna Alkaloid | 1 | Fentanyl Citrate Micronized | 400 | Prednisone | 5 |
| Benztropine Mesylate | 1 | Fesoterodine Fumarate | 4 | Prochlorperazine Malate | 5 |
| Brompheniramine Maleate | 4 | Flavoxate HCl | 300 | Prochlorperazine | 20 |
| Bupropion HCl | 300 | Fluvoxamine Maleate | 100 | Promethazine HCl | 25 |
| Captopril | 50 | Furosemide | 20 | Quinidine Sulfate Hydrate | 600 |
| Carbamazepine | 200 | Haloperidol | 2 | Ranitidine Bismuth Citrate | 800 |
| Carbinoxamine Maleate | 12 | Hydralazine HCl | 30 | Ranitidine HCl | 300 |
| Chlorpheniramine Maleate | 4 | Hydrocortisone | 10 | Risperidone | 2 |
| DL-Chlorpheniramine Maleate | 4 | Hydroxyzine HCl | 30 | Scopolamine Butylbromide | 30 |
| D-Chlorpheniramine Maleate | 90 | Hyoscyamine Sulfate Hydrate | 1 | Scopolamine HBr | 30 |
| Chlorpromazine HCl | 30 | Imipramine HCl | 25 | Scopolamine | 2 |
| Chlorthalidone | 25 | Isosorbide Dinitrate | 15 | Solifenacin Succinate | 5 |
| Cimetidine | 800 | Isosorbide Dinitrate Coated Gr. | 40 | Solifenacin Fumarate | 5 |
| Clemastine Fumarate | 1 | Isosorbide-5-Mononitrate | 40 | Solifenacin Tartrate | 5 |
| Clidinium Bromide | 8 | Isosorbide-5-Mononitrate S.R. Gr. | 40 | Thioridazine HCl | 75 |
| Clomipramine HCl | 25 | Isosorbide-5-Mononitrate Montan Glycol Wax Mixture 40% | 100 | Tolterodine L-Tartrate | 2 |
| Clozapine | 150 | Isosorbide Solution | 70 | Trazodone HCl | 150 |
| Codeine Phosphate Hydrate | 60 | Loperamide HCl | 6 | Trifluoperazine HCl | 5 |
| Colchicine | 1 | Loperamide Oxide Hydrate | 2 | Trihexyphenidyl HCl | 5 |
| Cyclobenzaprine HCl | 15 | Loxapine Succinate | 60 | Triprolidine HCl Hydrate | 8 |
| Cyproheptadine HCl Hydrate | 4 | Meclizine HCl Hydrate | 25 | Trospium Chloride | 40 |

The adequate daily dose for the elderly was determined for the anticholinergic agents that have been licensed in Korea.

Supplementary Table S4. The ICD-10 codes which were used to define each disease (group)

| Disease | ICD code |
| --- | --- |
| Diabetes mellitus | E10 E11 E12 E13 E14 |
| Hypertension | I10 |
| Myocardial infarction | I21 I22 I25 |
| Cardiovascular diseases | G45 G46 H34 I60 I61 I62 I63 I64 I65 I66 I67 I68 I69 |
| Dizziness | H81 H93 R42 |
| Sleep disorder | G47 F51 |
| Genitourinary diseases | N30 N31 N32 N39 N40 R32 R35 |
| Epilepsy | G40 G41 |
| Parkinson’s diseases | G20 G21 G22 G23 |
| Neuralgia | G53 M45 M46 M47 M48 M49 |
| Respiratory diseases | J06 J00 J10 J20 J30 J45 |
| Skin diseases | L20 L21 L23 L24 L30 L50 |

Abbreviation: ICD-10, Internationl Classification of diseases-10

**Supplementary Table S5.** **Other non-anticholinergic agents that can impair cognitive function (referenced by AGS Beers Criteria)**

| Amisulpride | Flurazepam | Paliperidone |
| --- | --- | --- |
| Aripiprazole | Flutoprazepam | Pinazepam |
| Blonanserin | Haloxazolam | Quazepam |
| Bromazepam | Lafutidine | Quetiapine |
| Bromperidol | Levomepromazine | Roxatidine |
| Brotizolam | Lithium | Sulpiride |
| Chlordiazepoxide | Loprazolam | Temazepam |
| Chlorprothixene | Lorazepam | Thiothixene |
| Clobazam | Melperone | Tiapride |
| Clonazepam | Mesoridazine | Tofisopam |
| Clorazepate | Mexazolam | Triazolam |
| Estazolam | Midazolam | Ziprasidone |
| Ethyl Loflazepate | Mosapramine | Zolpidem |
| Famotidine | Nemonapride | Zopiclone |
| Fludiazepam | Niperotidine | Zotepine |
| Fluocinolone | Nizatidine | Zuclopenthixol |
| Flupentixol | Nordazepam |  |
